# Current experimental methods to investigate the impact of specialized pro-resolving lipid mediators on Sjögren’s syndrome

**DOI:** 10.3389/fimmu.2022.1094278

**Published:** 2023-01-12

**Authors:** Harim T. dos Santos, Kihoon Nam, Diana Gil, Venkata Yellepeddi, Olga J. Baker

**Affiliations:** ^1^ Bond Life Sciences Center, University of Missouri, Columbia, MO, United States; ^2^ Department of Otolaryngology-Head and Neck Surgery, School of Medicine, University of Missouri, Columbia, MO, United States; ^3^ Department of Surgery, School of Medicine, University of Missouri, Columbia, MO, United States; ^4^ Department of Molecular Microbiology and Immunology, School of Medicine, University of Missouri, Columbia, MO, United States; ^5^ Department of Biological and Biomedical Engineering, College of Engineering, University of Missouri, Columbia, MO, United States; ^6^ Division of Clinical Pharmacology, Department of Pediatrics, School of Medicine, University of Utah, Salt Lake City, UT, United States; ^7^ Department of Molecular Pharmaceutics, College of Pharmacy, University of Utah, Salt Lake City, UT, United States; ^8^ Department of Biochemistry, University of Missouri, Columbia, MO, United States

**Keywords:** resolvins, immunology, mathematical modeling, cultures, mice, saliva, salivary glands

## Abstract

Sjögren’s syndrome is a chronic inflammatory autoimmune disease characterized by diminished secretory function of the exocrine glands. Although extensive investigation has been done to understand Sjögren’s syndrome, the causes of the disease are as yet unknown and treatments remain largely ineffective, with established therapeutic interventions being limited to use of saliva substitutes with modest effectiveness. A primary feature of Sjögren’s syndrome is uncontrolled inflammation of exocrine tissues and previous studies have demonstrated that lipid-based specialized pro-resolving mediators reduce inflammation and restores tissue integrity in salivary glands. However, these studies are limited to a single specialized pro-resolving lipid mediator’s family member resolvin D1 or RvD1 and its aspirin-triggered epimer, AT-RvD1. Consequently, additional studies are needed to explore the potential benefits of other members of the specialized pro-resolving lipid mediator’s family and related molecules (*e.g.*, additional resolvin subtypes as well as lipoxins, maresins and protectins). In support of this goal, the current review aims to briefly describe the range of current experimental methods to investigate the impact of specialized pro-resolving lipid mediators on Sjögren’s syndrome, including both strengths and weaknesses of each approach where this information is known. With this article, the possibilities presented by specialized pro-resolving lipid mediators will be introduced to a wider audience in immunology and practical advice is given to researchers who may wish to take up this work.

## 1 Introduction

Sjögren’s syndrome (SS) is a chronic inflammatory autoimmune disease characterized by diminished secretory function of the exocrine glands (1–9). It affects approximately 1% of the general population and up to 3% of people above the age of fifty, with women accounting for more than 90% of diagnosed cases (10, 11). Although extensive investigation has been done to understand the disease (12–14), the causes of SS are as yet unknown and treatments remain largely ineffective, with established therapeutic interventions being limited to use of saliva substitutes with modest effectiveness (15–17) and medications that provide only temporary relief (16). As such, development of alternative treatments to restore salivary gland (SG) functioning is critical.

A primary feature of SS is a systemic uncontrolled inflammation of exocrine tissues (18, 19) thought to be initiated by viral and bacterial infection together with the activation of susceptibility genes (20–24). This leads to an initial tissue damage followed by cytokines and chemokine release (25–28) as well as exaggerated antibody production by hyperactivated B cells (7, 18, 19), all of which can be detected on exocrine tissues and serum (29). When resolution mechanisms are working properly, neutrophils and M2 macrophages are able to clear the injury/infection site (30–33). However, when these mechanisms are absent, dead cells cannot be removed from the injury/infection site leading to production of autoantigens, elevations in chemokine and cytokine levels (34) that stimulate peripheral lymphocytes to bind to and infiltrate across the vascular endothelium into the SG (35–37), which leads to chronic immune responses, SG damage and dysfunction (38–41). To fully investigate the cell-specific mechanisms involved in the initiation of the inflammatory response, proven mouse models having SS-like features are required (42–45) to allow for identification of novel targets and approaches for inhibiting a futile systemic immune response and averting chronic inflammation (46–49).

**Figure 1:**
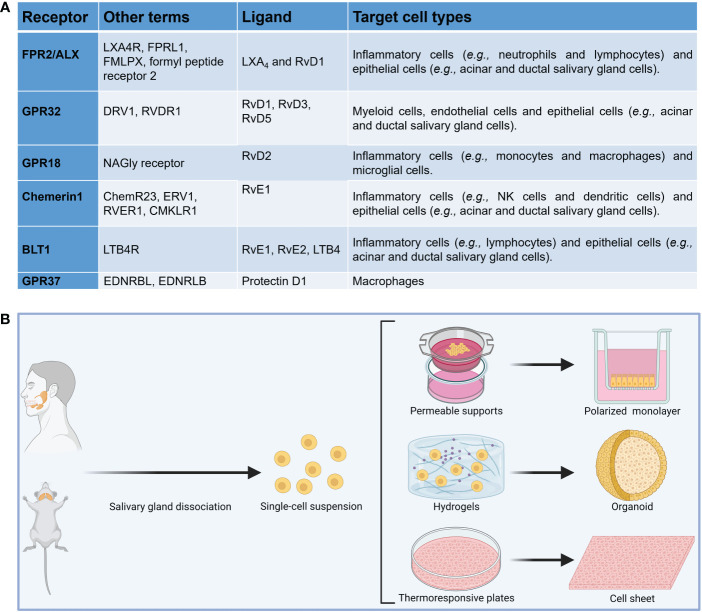
**(A)** Summary of SPM receptors, ligands and target cell types implicated in the resolution of inflammation. **(B)** Diagram showing common *in vitro* models to investigate the impact of specialized pro-resolving lipid mediators in salivary glands.

Resolution of inflammation is an actively regulated process mediated in part by a family of specialized pro-resolving lipid mediators (SPM) (50–54) which include resolvins, maresins, lipoxins and protectins as well as their aspirin-triggered (AT) forms, which are comparable in their properties to naturally occurring SPM (reviewed in (55–62), but have a longer half-life (58, 63). The SPM receptors belong to the G-protein coupled receptor (GPCR) family as detailed in **Figure 1A** (64–66). SPM and their AT forms present an intriguing alternative for treating inflammatory diseases by limiting uncontrolled inflammation in response to environmental challenges (67–73) while at the same time promoting its termination and likewise leading to tissue regeneration (74–81). They have been detected in human fluids (*e.g.*, saliva) as well as in animal models of infection and inflammation (82–87). Studies of SPM and AT forms within the SG have been largely confined to a single resolvin (AT-RvD1, one of many in the resolvin family) that has shown promise for treating hyposalivation. Specifically, previous studies demonstrated that AT-RvD1 reduces inflammation and restores tissue integrity in SG cells (88–91), AT-RvD1 biosynthetic and signaling pathways are active in mice and human SG (89–93), the progression of the SS-like features in NOD/ShiLtJ mice is halted using AT-RvD1, with mice treated systemically prior to disease onset displaying downregulation of pro-inflammatory cytokines, upregulation of anti-inflammatory molecules and intact saliva production (44, 94), similar results are obtained with AT-RvD1 treatment at disease onset and lack of the RvD1 receptor ALX/FPR2 leads to impaired innate (42) and adaptive immunity (33) in mouse submandibular glands (SMG), with such results derived within the SG being consistent with studies obtained using the full range of SPM in other organs (95–105). Despite the promise shown by SPM in general and AT-RvD1 in particular for treating SS, there are several obstacles that must be overcome for this treatment to be implemented in oral medicine. First, we only know the effects of RvD1 and AT-RvD1 within the SG (43, 44, 88, 91, 94), while other SPM that may contribute to resolution of inflammation in the SG are as yet unexplored. Next, there is a limited knowledge regarding how specific resolution mechanisms work in SG *in vivo* (106, 107) and still less is known in humans. Finally, researchers need to know the available options to study SPM in relation to autoimmunity; however, information as to the relative benefits and limitations of common experimental methods is not readily available in a single source. Together, an overarching review of the investigation models used to better understand the impact of SPM on SS is needed and for the purpose of this compilation we have divided the methods into three broad categories: *in vitro* studies, *in vivo* studies and mathematical models.

## 2 Methods to investigate SPM using *in vitro* models

The main eicosapentaenoic acid (EPA), docosahexaenoic acid (DHA) and arachidonic acid (AA)-based metabolomes operating during resolution of inflammation have been discovered and can provide insight into the mechanisms of inflammation resolution in multiple organs (65, 108). Applying these findings to SS, previous studies found that these metabolomes are present in mouse and human SG as well as saliva (109). Given the above, it is necessary to study spatio-temporal SPM biosynthesis and the upstream signaling mechanisms for EPA, DHA and AA-based metabolomes during resolution of inflammation in SS. Generally speaking, however, understanding of SPM upstream signaling mechanisms in SG lags behind that of downstream mechanisms in other organs (58–60, 103, 110–113) and, as earlier stated, the only SPM to have been studied extensively to date is RvD1 and its aspirin-triggered epimer (88–91, 94).

To investigate whether SPM biosynthesis is dysregulated *in vitro*, several models using freshly isolated cells from SS-like mouse models and SS human specimens can be compared to their healthy counterparts. These cells may be plated on permeable supports to allow monolayer formation (91, 114), scaffolds (*e.g.*, hydrogels) to form SG organoids (94, 115) or thermoresponsive plates to form cell sheets (116) (**Figure 1B**). Permeable supports will permit us to study apico-basal polarized SPM biosynthesis while scaffolds will help us to understand polarized SPM biosynthesis in a three-dimensional environment. Nevertheless, permeable supports assays have limitations, including staining steps that may wash off attached cells and unevenly distributed membrane pores; however, protocol optimization can minimize this limitation (117). Moreover, cell sheets will further our understanding of how SPM are produced in a more natural environment (*i.e.*, in the presence of extracellular matrix proteins and secretory granules), which are absent in the prior two models. A limitation of cell sheets is that the exact cell composition is still under investigation and future studies using RNA seq might be needed (118). Finally, the use of fresh human minor SG can verify results obtained with mouse models in humans; yet cell culture optimization is critical to ensure cells viability under *in vitro* conditions. Measuring SPM and their metabolites at various times points in each of these models using both ELISA (109) and targeted metabololipidomics (119), with results from each *in vitro* model analyzed and SPM biosynthesis among these complementary models, can provide an approximation of how SPM biosynthesis occurs *in vivo*. These experiments can use EPA, DHA and AA as substrates for SPM biosynthesis *in vitro* while also using ELISA and metabololipidomics in response to these lipids (109, 119).

SPM are generated in resolvin exudates *in vivo* and are a product of transcellular biosynthesis with human leukocytes and endothelial or epithelial cells (63, 65, 120), with sequential oxygenations by human 15-lipoxygenase (LOX) in humans or mouse ortholog 12/15-LOX and 5-LOX (121) involved in their biosynthesis. The first step in SPM biosynthesis involves release of ω-3 fatty acids from membrane phospholipids by PLA_2_, which is responsible for lipid release in mammalian cells (122, 123). The second step is transformation of ω-3 fatty acids to 17S-Hp forms by the enzymatic conversion through 15-LOX or 12/15-LOX forms (61, 63, 124), with the final step in SPM biosynthesis being release of 17S-Hp forms from epithelial or endothelial cells to the extracellular medium. 17S-Hp forms are then captured by adjacent cells and transformed into SPM by the action of the enzyme 5-LOX (61, 63, 124). In the presence of aspirin, the 15-LOX or 12/15-LOX activities in epithelial cells are transformed into cyclooxygenases by COX-2 activation (63). This leads to the formation of 17R-Hp forms, which is transformed to AT-forms by 5-LOX (63). In light of the above, it is important to measure expression of biosynthetic enzymes, determine their activities and quantify metabolite production in response to ω-3 fatty acids. Finally, for these studies it is critical to measure the impact of enzymes involved in SPM biosynthesis using mouse lines specific to each particular enzyme (*e.g.*, 5LOX^-/-^).

## 3 Methods to investigate SPM using *in vivo* models

Having expanded SPM mechanistic studies to additional morphological factors using the various *in vitro* models listed above, it is necessary to further isolate SPM mechanisms by studying their metabolomes *in vivo*, thereby tracking mechanisms originating in the SG and operating in conjunction with other organs throughout the body, as detailed below. Specifically, SPM biosynthesis and signaling pathways may be activated in response to a wide variety of internal and/or environmental stressors (31, 125–128), such that the use of multiple mouse models covering these various conditions can be utilized as follows: NOD/ShiLtJ mouse model for autoimmune disease (129–134), NOD.H-2h4 mouse expressing the major histocompatibility complex haplotype H-2K on the NOD mouse background, rendering the NOD.H-2h4 mouse susceptible to autoimmune-like features, including spontaneous thyroiditis and SS-like features, but not diabetes (135–137), surgically wounded mouse SMG for tissue injury (138–140) and LPS-challenged mouse model (42). Additionally, although the NOD mouse lines are accepted models for SS, use of the wounded and LPS mice facilitate comparisons between inflammation due to physical injury and infection, respectively. Together, these models allow for measurement of SPM production in plasma, saliva and SMG during both acute and chronic inflammation.

To further identify SPM downstream mechanisms, receptor signaling studies are necessary. For instance, the G protein-coupled ALX/FPR2 receptor for AT-RvD1 is effective in restoring secretory function in SS mouse SG (43, 44, 94), indicating that activation of this receptor diminishes the systemic immune response. Given the remarkable effects with AT-RvD1, it is important to investigate the downstream mechanisms that resolve chronic inflammation *via* ALX/FPR2 or through activation of additional SPM receptors using SS mouse models and test the effect of targeting SPM in SS mouse models to diminish systemic immune response. One such model is the NOD/ShiLtJ mouse that can be studied at five time points representing the pre-disease stage (4 wk), the early pre-clinical stage (8 wk), the early clinical phase of autoimmunity (12-16 wk) and the onset of clinical SS-like features characterized by secretory dysfunction (16-20 wk). At 8-16 wk, lymphocytic infiltration and upregulation of SS-associated pro-inflammatory cytokines are observed, while at 20 wk, anti-nuclear antibodies and anti-M3 autoantibodies are detected in sera of NOD/ShiLtJ mice together with decreased amylase activity (141–144). Together, the progressive changes in SS-like phenotype suggest that immune cell subpopulations mediate distinct aspects of the NOD mouse model’s autoimmune features and mimics SS clinical presentation by exhibiting hypergammaglobulinemia and elevated levels of immunoglobulin-secreting B lymphocytes (7, 18, 19). Previous studies demonstrated that systemic treatment with AT-RvD1 partially decreases lymphocytic infiltration of SMG and restores saliva secretion (43, 44, 88, 94); however, to completely eliminate lymphocytic infiltration in the SS mouse models, it would be necessary to determine whether a combination of SPM completely abolishes lymphocytic infiltration and diminished saliva secretion. Should such a response be noted, it would be beneficial to elucidate their downstream signaling mechanisms. Therefore, the investigator should identify the temporal changes in SS-like pathological endpoints following systemic treatment with a range of SPM, quantify alternations in inflammatory cells and their SPM receptor expression and identify changes in the formation of hematopoietic stem cells and lymphoid progenitor cells. In SS, epithelial cells adjacent to sites of inflammation display high levels of immunoactive proteins (145, 146), cell adhesion molecules (147–150) and proinflammatory mediators (25, 28, 151, 152), thus playing a role in regulation of immune cells. Given that SPM have been shown to reduce these events in a range of other tissues (153–157), contributions of SPM receptors to the autoimmune phenotype of SS mouse models should be investigated.

## 4 Methods to investigate SPM metabolism using mathematical models

**Figure 2:**
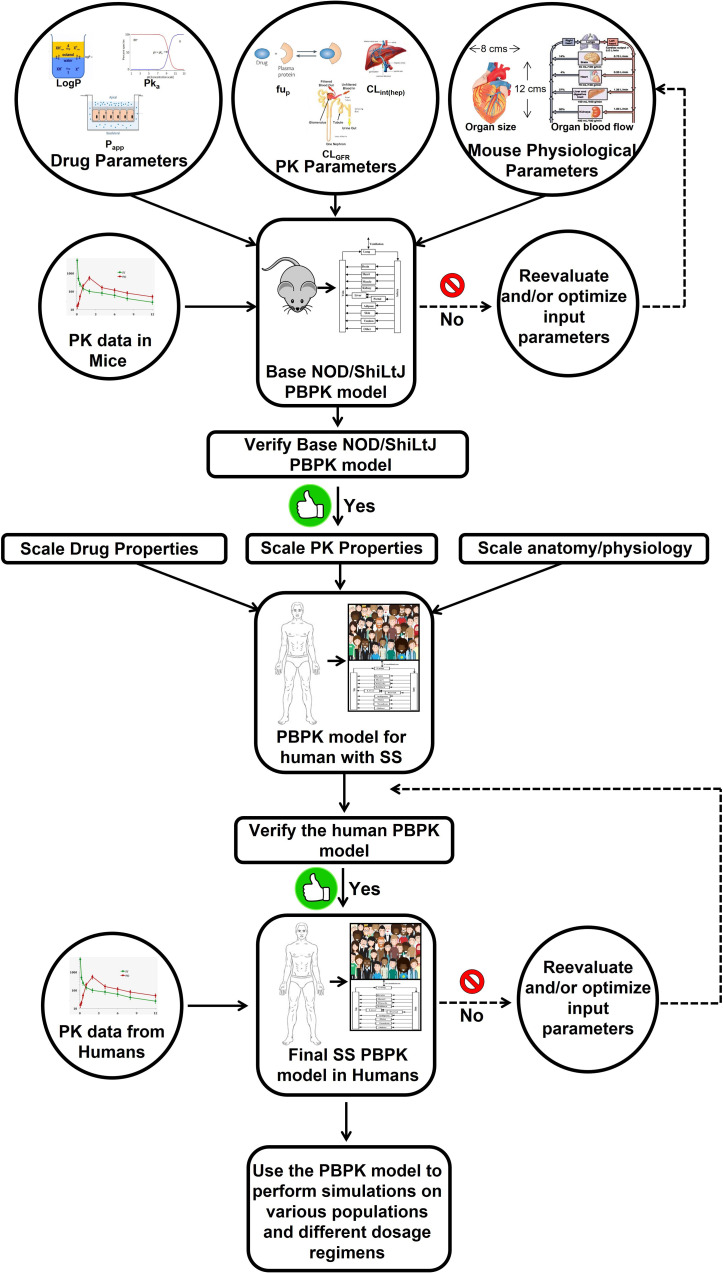
Workflow of PBPK model development for the specialized pro-resolving lipid mediator AT-RvD1. The PBPK model for human with Sjögren’s syndrome refers to the virtual population of the subjects whose anatomical physiological parameters are consistent with disease features. If the model-predicted data does not agree with the observed experimental data, a parameter identification and optimization will be performed using the default tools provided by the PK-Sim^®^ software to identify the parameter responsible for the lack of accurate predicts and then its value will be optimized using the observed data until a goodness of fit is achieved between predicted versus observed data. Although AT-RvD1 is the subject of the figure, the same model can be applied to other specialized pro-resolving lipid mediators. This image was adapted from (163). AT-RvD1: aspirin-triggered resolvin D1; PBPK, physiologically based pharmacokinetic; PK, pharmacokinetics; SS, Sjögren’s syndrome.

Mathematical models such as those presented below may be used as supplementary methods to inform study design using the principal methods already presented (*i.e.*, *in vivo* and *in vitro* studies), and in so doing the investigator can better focus their traditional lab work to better use resources (*i.e.*, time and lab materials) and also reduce the suffering of animals (*e.g.*, *in vitro* studies). These mathematical models using differential equations, virtual models of the systems to be studied, both in terms of the minimum effective treatment dose of a given drug as well as its likely path and interactions within the body. The payoff on such methods should be apparent – they provide an educated guess as to a starting point for dose-response studies, thereby resulting in a better chance of getting a desired result with fewer trials as compared to a literature review and/or trial and error alone. Likewise, the results derived from *in vitro* and *in vivo* studies subsequent to mathematical modeling can then be fed back into a revised and verified version of that model, based on these real-world inputs, to better inform future iterations of the investigative process. Recently, various types of mathematical models have been applied to the drug development process (158–162). Principal among them is pharmacokinetic (PK) modeling, which combines investigator-determined pharmacological parameters with the biological and physiological parameters derived from general experience and/or literature review to achieve a well-informed virtual mechanistic representation of that drug, thereby allowing for *a priori* simulation of expected concentration-time profiles (158–160). Furthermore, PK modeling enables the estimation of drug exposure, not only in plasma but also at the site of action, which may be difficult or impossible to measure experimentally (158–160). Given the utility of a well-validated PK model for estimating SPM concentrations *in vivo*, it is likewise necessary to produce a whole-body mouse PK model of SPM to be coupled with an earlier derived pharmacodynamics (PD) model. Such a PK/PD tandem allows the PD effects of SPM (*e.g.*, salivary flow as well as local and systemic inflammation binding to simulated plasma and SG SPM concentrations) to be quantified. The PK/PD model can then be validated by administering the target dose of SPM to a healthy mouse, sacrificing it and measuring its concentration within specified organs using liquid chromatography-tandem mass spectrometry (LC-MS/MS) as well as ELISA analyses (109, 119), thus establishing baseline values for future SPM studies (**Figure 2**). The target administration amount can then be introduced systemically at multiple time points, after which the degree of inflammatory resolution in the SG and the mechanisms by which this change has occurred should be determined. The validated mouse PK/PD effects obtained in mice can be extrapolated to humans by replacing the model input parameters for the mouse species with those of humans on the basis of a previously developed whole-body PK model of AT-RvD1 and extrapolation protocol for humans (163, 164), a process of model development that will need to be replicated for exploration of future SPM to be identified and explored in this context. However, the PK model has some limitations. First, the physicochemical and biological parameters used to build the mouse models are obtained either from the literature or estimated using algorithms and need to be confirmed experimentally. Second, in PK human models for RvD1 and AT-RvD1 data must be verified using results from prospective clinical studies. Finally, these models do not consider endogenous RvD1 which may influence the PK of exogenous administrated RvD1 and AT-RvD1.

## 5 Discussion

This review has been presented with the aim of promoting both the rationale and means to study SPM as a potential treatment for SS. The need to do so is based on the current lack of effective treatments and the methods for doing so, as detailed above, are numerous and ever expanding. Regarding *in vitro* studies, results should be interpreted with caution as SPM production may be different depending on growth and differentiation stages within the SG as well as the various culture conditions. Moreover, if the researcher finds SPM dysregulation to occur in human tissue cultures, studies should be confirmed multiple times and correlated with clinical data. As for *in vivo* models, previous studies indicated that endotoxin challenge temporally regulates lipid mediator production in human serum (50), where pro-inflammatory eicosanoids concentrations peak after 8 h and similar results could occur in SG. Also, SPM such as resolvins and lipoxins initially decreased after 2 h but are then elevated at 24 h. Therefore, a similar timing of SPM production may occur in *in vivo* models of inflammatory resolution (*i.e.*, wound healing (138–140) and innate immunity (165) models); however, dysregulation of SPM production in SS mouse model may occur during disease progression, as these mice do not resolve inflammation (1, 44, 46, 48, 166). With respect to virtual mathematical models (158–162), such techniques can provide a reasonable starting point for required total dosage to be administered while also eliminating the need for excessive animal usage, as would be the case should this estimate be derived by trial and error alone; however, a limitation of mathematical models is that the physicochemical and biological parameters used to build the RvD1 and AT-RvD1 PK/PD models are obtained either from data on from data, from the literature or estimated using algorithms (163, 164). Therefore, it is critical to keep in mind that all such estimates must be confirmed and thus validated using models *in vivo*. To expand on this work, future investigations may involve determining if activation of SPM receptors in SG epithelium and vascular SG endothelium diminishes SS autoimmune phenotype, identifying mechanisms by which SPM receptors diminish SG lymphocytic infiltration in SS mouse models and testing whether SPM activation in B and T cells diminishes SS phenotype. Finally, it is worth emphasizing yet again that the SPM family is vast and work has only been performed in relation to the SG with a small fraction of the potential candidates. It would therefore appear warranted to extend the range of SPM to be explored by repeating already performed studies with new SPM candidates and related molecules while also stretching this work to make use of emerging experimental techniques and technologies.

## Author contributions

HS, KN, DG, and VY wrote the manuscript and prepared figures. OB planned, wrote and overall supervised the preparation of the manuscript. All authors contributed to the article and approved the submitted version.
